# Expression of fourteen novel obesity-related genes in zucker diabetic fatty rats

**DOI:** 10.1186/1475-2840-11-48

**Published:** 2012-07-13

**Authors:** Peter M Schmid, Iris Heid, Christa Buechler, Andreas Steege, Markus Resch, Christoph Birner, Dierk H Endemann, Guenter A Riegger, Andreas Luchner

**Affiliations:** 1 Klinik und Poliklinik für Innere Medizin II, University of Regensburg, Regensburg, Germany; 2 Institut für Epidemiologie und Präventivmedizin, University of Regensburg, Regensburg, Germany; 3 Klinik und Poliklinik für Innere Medizin I, University of Regensburg, Regensburg, Germany; 4 Klinik und Poliklinik für Innere Medizin II, Franz-Josef-Strauss-Allee 11, University of Regensburg, 93042, Regensburg, Germany

**Keywords:** BDNF, ETV5, FAIM2, FTO, GNPDA2, KCTD15, LYPLAL1, MCR4, MTCH2, NEGR1, NRXN3, TMEM18, SEC16B, TFAP2B, ZDF-rats

## Abstract

**Background:**

Genome-wide association studies (GWAS) are useful to reveal an association between single nucleotide polymorphisms and different measures of obesity. A multitude of new loci has recently been reported, but the exact function of most of the according genes is not known. The aim of our study was to start elucidating the function of some of these genes.

**Methods:**

We performed an expression analysis of fourteen genes, namely BDNF, ETV5, FAIM2, FTO, GNPDA2, KCTD15, LYPLAL1, MCR4, MTCH2, NEGR1, NRXN3, TMEM18, SEC16B and TFAP2B, via real-time RT-PCR in adipose tissue of the kidney capsule, the mesenterium and subcutaneum as well as the hypothalamus of obese Zucker diabetic fatty (ZDF) and Zucker lean (ZL) rats at an age of 22 weeks.

**Results:**

All of our target genes except for SEC16B showed the highest expression in the hypothalamus. This suggests a critical role of these obesity-related genes in the central regulation of energy balance. Interestingly, the expression pattern in the hypothalamus showed no differences between obese ZDF and lean ZL rats. However, LYPLAL1, TFAP2B, SEC16B and FAIM2 were significantly lower expressed in the kidney fat of ZDF than ZL rats. NEGR1 was even lower expressed in subcutaneous and mesenterial fat, while MTCH2 was higher expressed in the subcutaneous and mesenterial fat of ZDF rats.

**Conclusion:**

The expression pattern of the investigated obesity genes implies for most of them a role in the central regulation of energy balance, but for some also a role in the adipose tissue itself. For the development of the ZDF phenotype peripheral rather than central mechanisms of the investigated genes seem to be relevant.

## Background

By now, overweight and obesity have become a major public health problem in the industrialized world and also in developing countries. Both result in an increased morbidity and mortality, not to mention the financial costs for the health-care systems. According to the World Health Organisation (WHO) in 2005 worldwide at least 400 million adults were obese and this count is expected to rise to 700 million until 2015 (http://www.who.int/gho/ncd/risk_factors/overweight/en/). Thereby, lifestyle changes with an increased intake of energy-dense foods and a trend to decreased physical activity are fundamental causes for this rise to epidemic proportions, but heritability studies provide evidence for a notable genetic involvement [1,2].

Genome-wide association studies (GWAS) are excellently qualified to reveal single genetic polymorphisms associated with human obesity, but they allow no conclusions about the function of the affected genes. Since recent GWAS [3-11] in adults could find different genetic loci located next to or in so far partly unknown genes, now the physiologic role of most of these genes has to be elucidated.

The aim of our study was to shed some light in the role of fourteen recently described genes, namely BDNF, ETV5, FAIM2, FTO, GNPDA2, KCTD15, LYPLAL1, MCR4, MTCH2, NEGR1, NRXN3, TMEM18, SEC16B, and TFAP2B, which were associated with different measures of human obesity. Thereby, as a first step it is reasonable to identify tissues, in which those genes are expressed, and to reveal differences in the expression levels between obese and lean subjects. Therefore our study was planned as a descriptive expression analysis of these obesity-related genes in an animal model of diabetes mellitus type 2 and obesity, the ZDF rat, in comparison to their lean normoglycemic littermate, the ZL rat.

Some of the obesity-genes are already known to regulate energy homeostasis and for some others it is shown that they are expressed in the central nervous system [11] and may influence energy expenditure centrally in the hypothalamus. For other genes functions apart from the regulation of energy homeostasis are described and for some genes their function is totally unknown. Since the adipose tissue is not only a simple energy storage, but also a key regulator of body weight [12,13], it is also a possible location of action for these obesity-related genes. Thereby, it is important to differentiate between adipose tissues of different origin as it is known that especially mesenterial adipose tissue harbors a high risk for comorbidities like diabetes mellitus type 2 and atherosclerosis [14]. Therefore the gene expression was measured via real-time RT-PCR in the hypothalamus as regulatory center of energy balance and food intake [15] and in the adipose tissue of the mesenterium, the subcutaneum and the kidney capsule.

## Methods

### Animal experiment

The study was approved by the local committee on animal research and is in accordance with the “Guide for the Care and Use of Laboratory Animals’ published by the US National Institutes of Health. The EU legislation for animal care (86/609/CEE) was applied. Six male Zucker diabetic fatty (ZDF) rats (fa/fa) and six male Zucker lean rats (fa/-) (ZL) were studied from an age of 13 weeks until an age of 24 weeks. The animals were individually housed on a 12-hour dark/12-hour light cycle. They were fed a Purina 5008 rat chow containing 23% protein, 6.5% fat, 58.5% carbohydrates, 4% fiber and 8% ash. Rats received tap water ad libitum. In week 14 and 22 systolic blood pressure was assessed by tail cuff method using an automated cuff inflator-pulse detection system (CODA2 Multi-Channel, Computerized, EMKA TECHNOLOGIES, Paris, France). At the same timepoints the animals were weighted and blood glucose was measured after 6 hours fasting (ACCU-CHEK Sensor, Roche GmbH, Mannheim, Germany). Animals were sacrificed at 24 weeks of age and we prepared the adipose tissue of the mesenterium (mesenterial fat, MF), the kidney capsule (kidney fat, KF) and subcutaneum (subcutaneous fat, SF) as well as the hypothalamus (HT) of the six ZDF and ZL rats. Serum levels of leptin and adiponectin as marker of obesity were determined via ELISA according to the manufacturers instructions (B-Bridge International, Inc., 5 Mountain View, USA).

### Expression analysis via real-time RT-PCR

The fourteen target genes were chosen according to the results of recent GWAS and metaanalysis of GWAS [4-11], in which they were found to be significantly associated with different measures of obesity. The gene expression was measured in three different adipose tissues (KF (kidney fat), SF (subcutaneous fat) and MF (mesenterial fat)) and the hypothalamus (HT) by using the real-time RT-PCR technique. Therefore total RNA from the different tissues was extracted using the RNeasy kit (Quiagen, Hilden, Germany) according to the manufacturers instructions. A DNase digestion step was included. For first-strand cDNA synthesis, 1 μg total RNA was reverse transcribed with 1U MMLV Reverse Transcriptase, 1 μg Random Primer, 1 mM deoxynucleotide triphosphate mixture, 1 μl recombinant RNasin ® ribonuclease inhibitor and transcription buffer with 5 mM MgCl2 in a final volume of 10 μl (all from Promega, Mannheim, Germany). The reaction mixture was incubated at 37°C for 60 min, followed by heat inactivation of the enzyme at 95°C for 5 min. After cooling on ice for 5 min, the cDNA was stored at −20°C until further use. Real-time RT-PCR detection of the target genes and beta-actin as housekeeping gene was perfomed using the AbiPrism 7900 TaqMan (Applied Biosystems, Foster City; CA, USA). Primers and probes (Table 1) were used according to the manufacturer’s protocol (Applied Biosystems). The expression levels of the target mRNA were normalized to beta-actin using the DeltaCt method. Parallelism of amplification curves of the target and control was confirmed.

**Table 1:** Primer and probes

***Gene symbol***	***Gene name***	***Applied biosystems*** ***assay ID***
Actb	Beta Actin	Rn00667869_m1
BDNF	Brain derived neurotrophic factor	Rn02531967_s1
ETV5	Ets variant 5	Rn00465814_g1
FAIM2	Fas apoptotic inhibitory molecule 2	Rn01532650_m1
FTO	FTO	Rn01538183_m1
GNPDA2	Glucosamine-6-phosphate deaminase 2	Rn01413702_m1
LYPLAL1	Lysophospholipase-like 1	Rn01411856_g1
MC4R	Melanocortin 4 receptor	Rn01491866_s1
MTCH2	Mitochondrial carrier homolog 2	Rn01013168_m1
NRXN3	Neurexin 3	Rn00587546_m1
NEGR1	Neuronal growth regulator 1	Rn00572380_m1
KCTD15	Potassium channel tetramerisation domain containing 15	Rn01458149_m1
SEC16B	SEC16 homolog B	Rn00585728_m1
TFAP2B	Transcription factor AP-2 beta	Rn01511928_m1
TMEM18	Transmembrane protein 18	Rn01473465_g1

### Statistical analysis

The means of every measurement for the biometric data of ZL and ZDF rats as well as the means of the expression data from the real-time RT-PCR were calculated for each tissue sample and animal group. Groups were compared using the Students *t* test or one-way ANOVA as appropriate. A *p*-value of <0.05 was considered to be significant. The biometric data are presented as means ± SEM, the expression data are depicted in graphics with an arbitrary logarithmic y-axis as means + StdDev.

## Results and discussion

### Biometric data of ZL and ZDF rats

In Table 2 the biometric data of ZL and ZDF rats at an age of 22 weeks are summarized. As expected, ZDF rats demonstrated diabetes mellitus type 2 with a significantly elevated blood glucose, while the ZL rats were normoglycemic. The blood pressure was lower in the ZDF rats in terms of an advanced stage of disease. Surprisingly, the ZL rats weighted significantly more than the diabetic ZDF rats, but that was due to a better growth during the animal experiment, which was represented in a significantly greater tibia length in ZL rats. However the ZDF rats apparently had a different body composition with a smaller size and a higher body fat portion. This is also reflected by significantly higher serum levels of leptin and lower serum levels of adiponectin in ZDF rats.

**Table 2:** Biometric data of ZL and ZDF rats at an age of 22 weeks

Parameters	Week 22		
	ZL (n = 6)	ZDF (n = 6)	p
Blood glucose [mg/dl]	87 ± 1	393 ± 19	<0.001
SBP [mmHg]	119 ± 5	99 ± 2	0.003
Body weight [g]	405 ± 9	376 ± 4	0.01
Tibia length [cm]	4.5 ± 0.04	4.3 ± 0.03	0.008
Leptin [ng/ml]	10.3 ± 0.6	15.4 ± 1.6	0.013
Adiponectin [μg/ml]	23.0 ± 1.8	15.7 ± 0.6	0.003

Values are mean ± SEM. SBP indicates systolic blood pressure.

### Genes with a known role in regulation of energy homeostasis

**Figure 1:**
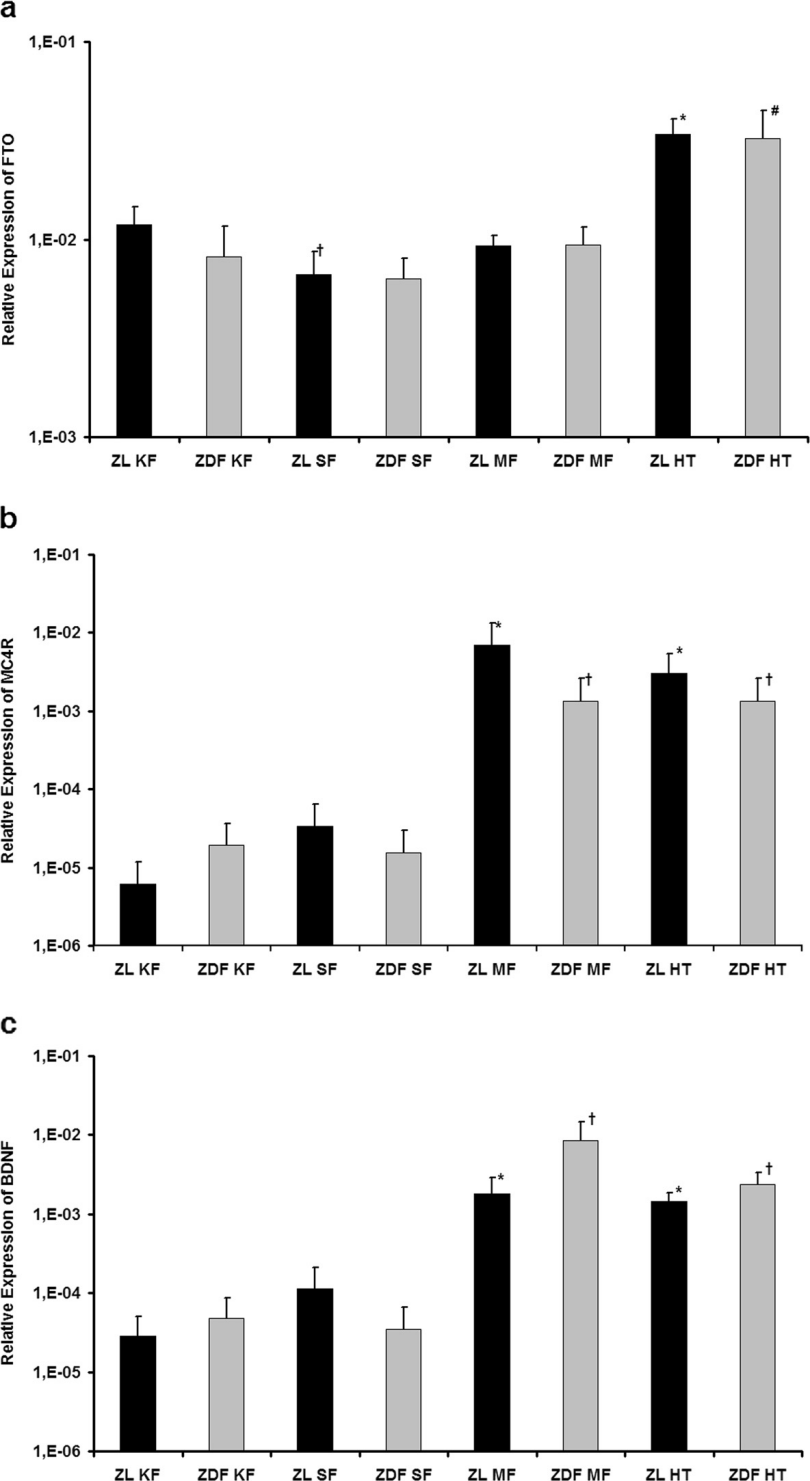
**Expression of genes with a known role in regulation of energy homeostasis.a)** Relative expression of FTO. ^*^p < 0.05 vs. ZL KF, ZL SF and ZL MF; † *p* < 0.05 vs. ZL KF, ZL MF and ZL HT; # *p* < 0.05 vs. ZDF KF, ZDF SF and ZDF MF. **b)** Relative expression of MC4R. ^*^p < 0.05 vs. ZL KF, ZL SF and ZL MF; † *p* < 0.05 vs. ZDF KF, ZDF SF and ZDF MF. **c)** Relative expression of BDNF. ^*^p < 0.05 vs. ZL KF and ZL SF; † *p* < 0.05 vs. ZDF KF and ZDF SF.

***FTO*** (fat mass and obesity associated, Figure 1a) was the first gene of the GWAS era, which was found to be associated with obesity [16], and therefore is one of the best investigated obesity-gene [17]. It is highly expressed in the arcuate nucleus of the hypothalamus and is supposed to have an anorexigenic function since an overexpression of FTO in the hypothalamus decreases and a knock-down of FTO increases food intake [18]. In our study the greatest amount of FTO mRNA in both animals was detected in the hypothalamus with significant differences to the other tissues (ZL and ZDF: *p* < 0.001 for HT vs KF, SF and MF). Only in ZL rats the expression in subcutaneous fat was significantly lower than in kidney and mesenterial fat (*p* = 0.001 for SF vs KF, *p* = 0.037 for SF vs MF), in ZDF rats the expression in the fat tissues was equally high. Significant changes between the animal groups for a single tissue could not be detected.

***MC4R*** (melanocortin 4 receptor, Figure 1b) as part of the central melanocortin system is highly expressed in the hypothalamus and mutations in the gene are thought to be a reason for obesity [19]. α-melanocyte stimulating hormone binds to MC4R in the hypothalamus, which leads to an enhancement of anorexigenic and an inhibition of orexigenic stimuli [20]. MC4R knock-out mice show the phenotype of the so called melanocortin obesity syndrome consisting of hyperphagia, hypometabolism, hyperinsulinemia and increased linear growth [21,22]. Generally, the central melanocortin system is accepted to be a key regulator of energy homeostasis and food intake, it affects body composition and diverse signals for the regulation of energy homeostasis like leptin interact with it [23]. The expression of MC4R showed the highest levels in the hypothalamus and mesenterial fat of ZL as well as ZDF rats. In kidney and subcutaneous fat the amount was significantly lower than in the hypothalamus and mesenterial fat (ZL: *p* < 0.001 for MF vs. KF, *p* = 0.001 for MF vs. SF, *p* < 0.001 for HT vs. KF, *p* = 0.002 for HT vs. SF; ZDF: *p* = 0.007 for MF vs. KF, *p* = 0.009 for MF vs. SF, *p* < 0.001 for HT vs. KF and SF). Comparing each single tissue between ZL and ZDF rats no significant difference could be found.

**BDNF** (brain derived neurotrophic factor, Figure 1c) as nerve growth factor is widely distributed throughout the whole brain [24]. Recently, BDNF was also suggested to be an important effector of the melanocortin system since BDNF infusion in the brain suppressed hyperphagia and weight gain in MC4R deficient mice [25]. For BDNF the expression level was similar high in the hypothalamus and mesenterial fat of ZL and ZDF rats each with significant differences to the other fat tissues (ZL and ZDF: *p* < 0.001 for HT vs. KF and SF, *p* < 0.001 for HT vs. MF). A significant difference between kidney fat and subcutaneous fat was not detected in both animal groups. Also, there were no significant differences between ZL and ZDF rats for a special tissue.

In summary, our data support the known role of these genes in the central regulation of energy balance even in ZL and ZDF rats, but the high expression of MC4R and BDNF in the mesenterial fat suggests also a possible direct action of the melanocortin system in the adipose tissue.

### Genes with a known neuronal expression

**Figure 2:**
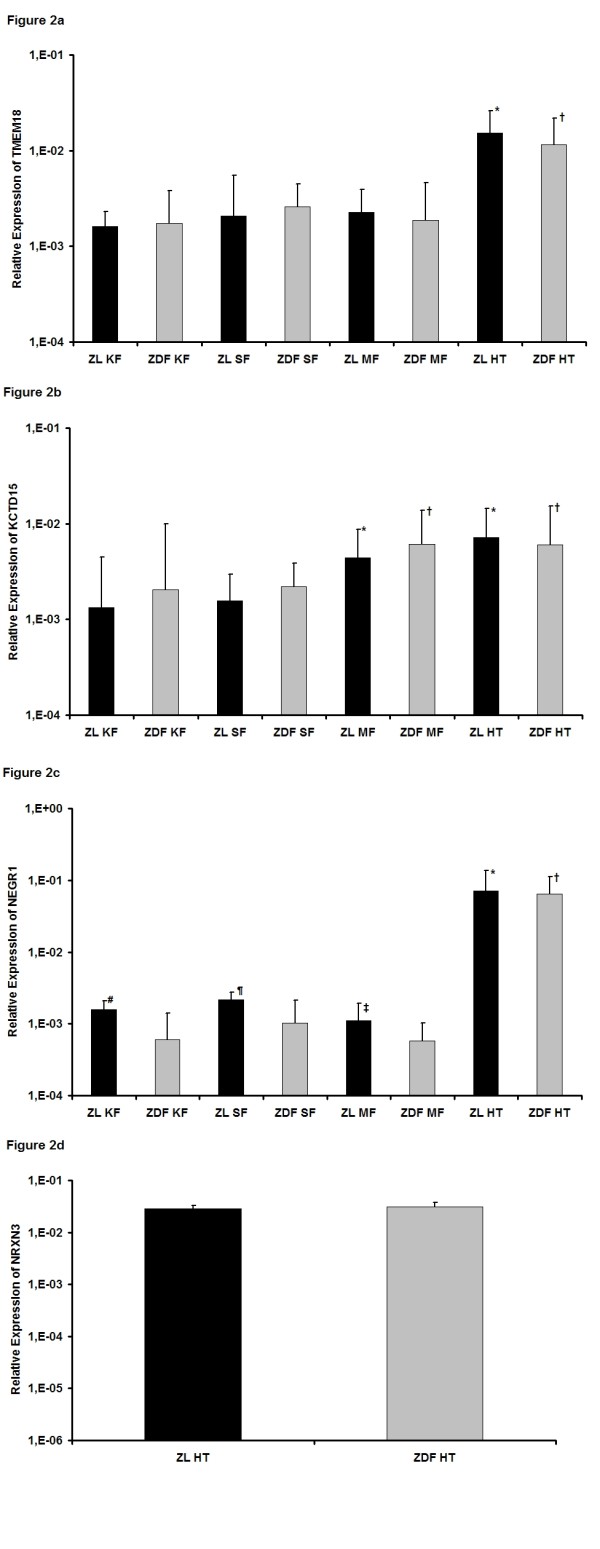
**Expression of genes with a known neuronal expression.a)** Relative expression of TMEM18. ^*^p < 0.05 vs. ZL KF, ZL SF and ZL MF; † *p* < 0.05 vs. ZDF KF, ZDF SF and ZDF MF. **b)** Relative expression of KCTD15. ^*^p < 0.05 vs. ZL KF and ZL SF; † *p* < 0.05 vs. ZDF KF and ZDF SF. **c)** Relative expression of NEGR1. ^*^p < 0.05 vs. ZL KF, ZL SF and ZL MF; † *p* < 0.05 vs. ZDF KF, ZDF SF and ZDF MF; # *p* < 0.05 vs. ZDF KF; ¶ *p* < 0.05 vs. ZDF SF; ‡ *p* < 0.05 vs. ZDF MF. **d)** Relative expression of NRXN3. No detectable mRNA-amount in adipose tissues.

***TMEM18*** (transmembrane protein 18, Figure 2a) was recently found to be remarkably conserved across different species and to be expressed in many brain sites without changes between feeding-related mouse models [26]. The expression pattern of TMEM18 in ZL and ZDF rats was similar without significant differences between each single tissues of the two animal groups. The highest amount of TMEM18 mRNA was found in the hypothalamus of both animals with significant differences to the different fat tissues (ZL and ZDF: *p* < 0.001 for HT vs KF, SF and MF).

***KCTD15*** (potassium channel tetramerisation domain containing 15, Figure 2b) is a BTB domain-containing protein and inhibits neural crest induction [27]. The highest quantitiy of KCTD15 mRNA was found for ZL and ZDF rats in mesenterial fat and the hypothalamus. Herein, the quantity was significantly higher than in kidney and subcutaneous fat (ZL: *p* < 0.001 for MF and HT vs KF and SF; ZDF: *p* = 0.008 for MF vs KF, *p* = 0.012 for MF vs SF, *p* = 0.009 for HT vs KF, *p* = 0.013 for HT vs SF). Significant differences for each single tissues between the two animal groups were not detected.

***NEGR1*** (neuronal growth regulator 1, neurotractin, Figure 2c) seems to play an important role in neuronal outgrowth during neurogenesis [28,29]. For both animal groups the quantity of NEGR1 mRNA was highest in the hypothalamus (ZL and ZDF: *p* < 0.001 for HT vs. KF, SF and MF). Interestingly, there were also significant differences between all three adipose tissues of ZL and ZDF rats. In kidney, subcutaneous and mesenterial fat the expression was significantly higher in ZL than in ZDF rats (KF: *p* = 0.002 for ZL vs. ZDF, SF: *p* = 0.027 for ZL vs ZDF, MF: *p* = 0.013 for ZL vs. ZDF).

***NRXN3*** (neurexin 3, Figure 2d) seems to play an important role in psychiatric disorders and recently it was also found to be expressed in other tissues like the heart [30]. The only tissue, in which mRNA of NRXN3 was detectable, was the hypothalamus of ZL and ZDF rats with no significant differences between the two animal groups.

Summing up, for TMEM18, KCTD15, NEGR1 and NRXN3 a neuronal expression is known and therefore a potential central effect on energy homeostasis is possible, but so far not yet described. Our data are in line with previous findings of the central expression of these genes. Recently, for NEGR1 additionally a differential expression between obese and lean siblings in subcutaneous fat was described and a central function in an obesity-related transcript network was suggested [31]. In our work, we could also show significant changes between obese and lean rats not only in the subcutaneous fat, but also in the kidney and mesenterial fat. This implicates a possible role of NEGR1 in the regulation of obesity in the adipose tissue itself and especially in the development of the ZDF phenotype.

### Genes with functions so far apart from the regulation of energy homeostasis

**Figure 3:**
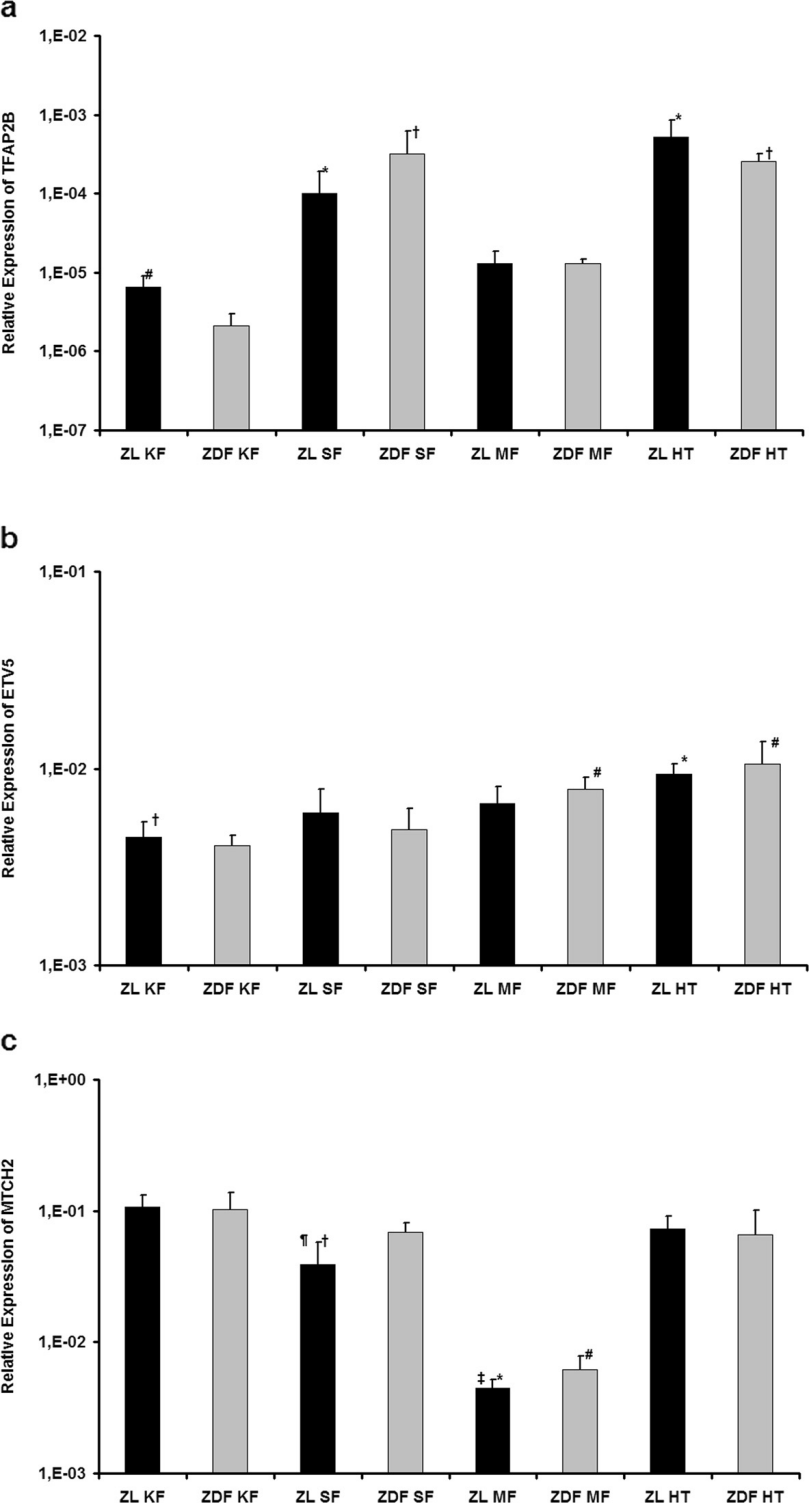
**Expression of genes with functions so far apart from the regulation of energy homeostasis.a)** Relative expression of TFAP2B. ^*^p < 0.05 vs. ZL KF and ZL MF; † *p* < 0.05 vs. ZDF KF and ZDF MF; # *p* < 0.05 vs. ZDF KF. **b)** Relative expression of ETV5. ^*^p < 0.05 vs. ZL KF, ZL SF and ZL MF; † *p* < 0.05 vs. ZL HT and ZL MF; # *p* < 0.05 vs. ZDF KF and ZDF SF. **c)** Relative expression of MTCH2. ^*^p < 0.05 vs. ZL KF, ZL MF and ZL HT; † *p* < 0.05 vs. ZL KF and ZL HT; # *p* < 0.05 vs. ZDF KF, ZDF SF and ZDF HT; ¶ *p* < 0.05 vs. ZDF SF; ‡ *p* < 0.05 vs. ZDF MF.

Mutations of ***TFAP2B*** (transcription factor activating enhancer binding protein 2 beta, Figure 3a) are known to cause the Char syndrome [32,33]. But recent studies could also show an expression in visceral and subcutaneous fat without a correlation to the body mass index or risk of diabetes mellitus type 2 in humans [34]. However, a negative correlation with the expression of adiponectin and leptin was found, which suggests that TFAP2B may regulate the expression of adipokines [34,35]. In our study the expression pattern of TFAP2 was similar between ZL and ZDF rats, each with highest expression in the hypothalamus and subcutaneous fat (ZL: *p* < 0.001 for HT vs. KF and MF, *p* = 0.003 for SF vs. KF, *p* = 0.021 for SF vs. MF; ZDF: *p* < 0.001 for HT vs. KF and MF, *p* < 0.001 for SF vs. KF and MF). In ZL rats the difference between hypothalamus and subcutaneous fat (*p* = 0.055) and in ZDF rats the difference between kidney and mesenterial fat (*p* = 0.051) did barely not reach the significance level. Comparing each single tissue between ZL and ZDF rats a significant higher expression level was only found in the kidney fat for lean animals. So, our results confirm the expression of TFAP2B in adipose tissues especially the subcutaneous fat, where it was equally high expressed as in the hypothalamus. Additionally, we could find that the expression of TFAP2B was not different between obese ZDF and lean ZL rats in the mesenterial and subcutaneous fat, but was significantly lower in the kidney fat of ZDF rats.

***ETV5*** (ets variant 5, Figure 3b) was until now mainly known to be an essential transcription factor for the establishment of the spermatogonial stem cell pool and its subsequent self-renewal and maintenance [36]. The expression pattern in our study of ZL and ZDF rats was quite similar without significant differences between the animal models for each tissue. Overall the highest expression was detected in the hypothalamus. In ZL rats the difference between hypothalamus and the adipose tissues was significantly (*p* < 0.001 for HT vs. KF, *p* = 0.007 for HT vs. SF, *p* = 0.037 for HT vs. MF) and also the expression in mesenterial fat was higher than in kidney fat (*p* = 0.018). For ZDF rats the amount of ETV5 mRNA in the hypothalamus was significantly greater than in the kidney fat and subcutaneous fat (*p* < 0.001 for HT vs. KF and SF), but slightly missed the significance level versus mesenterial fat (*p* = 0.058). In mesenterial fat it was significantly higher expressed than in kidney and subcutaneous fat (*p* < 0.001 for MF vs. KF, *p* = 0.005 for MF vs. SF). Herewith, our results reveal a so far not described high expression of ETV5 in the hypothalamus, which suggests a possible role in the central regulation of energy balance.

***MTCH2*** (mitochondrial carrier 2, Figure 3c) is a mitochondrial membrane protein and seems to regulate cell proliferation and apoptosis [37,38]. Lately, a high expression of MTCH2 was described in adipose tissue. It could be shown that the expression was higher in subcutaneous fat than in visceral fat and that the expression of MTCH2 was higher in obese than in lean women [39]. In our investigation the expression of MTCH2 was lowest in mesenterial fat of ZL and ZDF rats (ZL and ZDF: *p* < 0.001 for MF vs. KF, SF and HT). In ZDF animals the expression in the other three tissues was similar high, only in ZL rats the expression in the subcutaneous fat was lower than in the kidney fat and the hypothalamus (*p* < 0.001 for SF vs. KF, *p* = 0.001 for SF vs HT). In comparison of each single tissue between lean and obese animals the expression of MTCH2 in the subcutaneous (*p* = 0.017) and the mesenterial fat (*p* = 0.014) was significantly higher in ZDF rats. Therewith, our data are in line with aforementioned studies showing a higher expression of MTCH2 in the subcutaneous fat than in mesenterial fat und also finding a higher expression in obese ZDF rats than in lean ZL rats. However, a new finding is the high expression in the hypothalamus of both animal groups. Therefore MTCH2 may play an important role in central and peripheral regulation of obesity.

### Genes with unknown functions

The function of the GWAS obesity-related genes GNPDA2, FAIM2 and LYPLAL1 is to our knowledge mainly unkown and we report here the first expression data for these genes in comparison between obese and lean animals.

**Figure 4:**
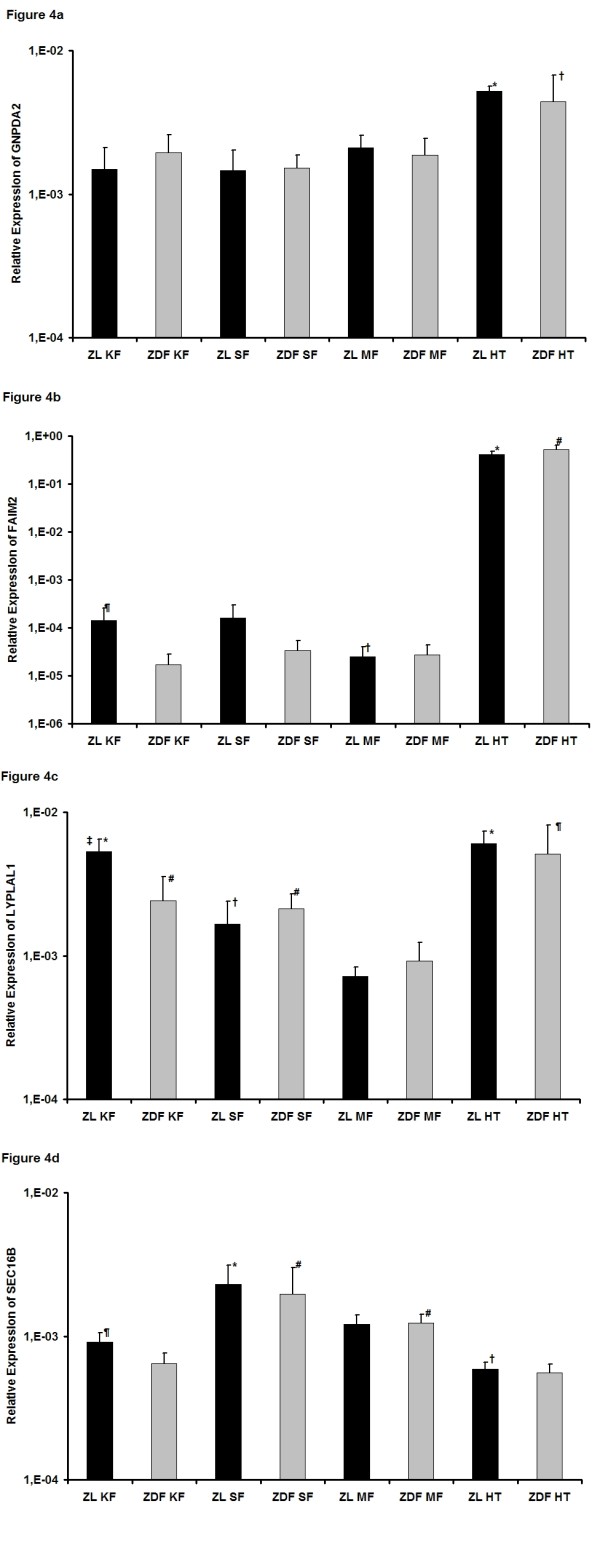
**Expression of genes with unknown functions.a)** Relative expression of GNPDA2. ^*^p < 0.05 vs. ZL KF, ZL SF and ZL MF; † *p* < 0.05 vs. ZDF KF, ZDF SF and ZDF MF. **b)** Relative expression of FAIM2. ^*^p < 0.05 vs. ZL KF, ZL SF and ZL MF; † *p* < 0.05 vs. ZL KF, ZL MF and ZL HT; # *p* < 0.05 vs. ZDF KF, ZDF SF and ZDF MF; ¶ *p* < 0.05 vs. ZDF KF. **c)** Relative expression of LYPLAL1. ^*^p < 0.05 vs. ZL SF and ZL MF; † *p* < 0.05 vs. ZL KF, ZL MF and ZL HT; # *p* < 0.05 vs. ZDF MF and ZDF HT; ¶ *p* < 0.05 vs. ZDF KF, ZDF SF and ZDF MF; ‡ *p* < 0.05 vs ZDF KF. **d)** Relative expression of SEC16B. ^*^p < 0.05 vs. ZL KF, ZL MF and ZL HT; † *p* < 0.05 vs. ZL KF and ZL MF; # *p* < 0.05 vs. ZDF MF and ZDF HT; ¶ *p* < 0.05 vs. ZDF KF.

The expression of ***GNPDA2*** (glucosamine 6 phosphate deaminase 2, Figure 4a) was for both animal models highest in the hypothalamus with significant differences to the fat tissues (ZL: *p* < 0.001 for HT vs. KF, SF and MF; ZDF: *p* = 0.008 for HT vs. KF, *p* = 0.001 for HT vs. SF, *p* = 0.006 for HT vs MF). The expression in kidney, subcutaneous and mesenterial fat was at similar levels with no differences between the ZL and ZDF rats.

For ***FAIM2*** (fas apoptotic inhibitory molecule 2, Figure 4b) the highest expression was observed for obese and lean animals in the hypothalamus with significant differences to the various fat tissues (ZL and ZDF: *p* < 0.001 for HT vs. KF, SF and MF). Whereas for ZDF rats the expression in the fat tissues was at the same level, it was significantly lower in mesenterial fat of ZL rats in comparison to the other fat tissues (*p* = 0.023 for MF vs. SF, *p* = 0.032 for MF vs. KF). Comparing each single tissues between both animal groups only in kidney fat a significantly decrease was detected in ZDF rats (*p* = 0.021).

Looking at ZL rats, the lowest expression of ***LYPLAL1*** (lysophospholipase like 1, Figure 4c) was found in mesenterial fat with significant differences to all other tissue samples (*p* <0.001 for MF vs. SF, KF and HT). Highest levels were seen in the hypothalamus and kidney fat (*p* < 0.001 for HT vs. SF, *p* < 0.001 for KF vs. SF). In ZDF rats the expression pattern was quite similar, only the expression in kidney fat was significantly lower than in ZL rats (*p* = 0.017), which resulted in a similar quantity to that of subcutaneous fat (*p* = 0.026 for MF vs. SF, *p* = 0.012 for MF vs. KF, *p* < 0.001 for MF vs HT, *p* = 0.020 for HT vs SF, *p* = 0.045 for HT vs. KF).

For ***SEC16B*** (SEC16 homolog B, Figure 4d) in ZL rats the highest expression was found in the subcutaneous fat with significant differences to the other tissues (*p* < 0.001 for SF vs. MF, KF and HT) and lowest in the hypothalamus with significant differences to the kidney and mesenterial fat (*p* = 0.008 for HT vs KF, *p* < 0.001 for HT vs. MF). In ZDF rats the pattern was quite similar (*p* < 0.001 for SF vs. KF and HT, *p* = 0.003 for MF vs. HT, *p* = 0.011 for MF vs KF), although the difference between subcutaneous and mesenterial fat slightly failed the significance level (*p* = 0.059). In the kidney fat the expression in ZL rats was significantly higher than in the ZDF rats (*p* = 0.010).

Taking together, whereas the high expression of these genes with unknown function in the hypothalamus again suggests a role in central energy regulation, LYPLAL1 with its notable expression in the kidney fat and the differences between ZDF and ZL rats implicates also a role in the adipose tissue itself. SEC16B was the only investigated gene that showed the highest expression not in the hypothalamus. Its function is mainly unknown, but it was highest expressed in the subcutaneous fat alone, which indicates a possible role in the peripheral regulation of obesity.

In summary, our results show that all of the fourteen target genes are expressed in the hypothalamus and in the investigated adipose tissues. The only exception was NRXN3, which was exclusively expressed in the hypothalamus and not in the adipose tissues. Likewise, TMEM18, NEGR1, GNPDA2, FTO, FAIM2 and ETV5 showed the highest expression in the hypothalamus. The highest expression for MC4R, KCTD15, BDNF, LYPLAL1, MTCH2 and TFAP2B was also observed in the hypothalamus, but the level was similar high for MC4R, KCTD15 and BDNF in the mesenterial fat, for LYPLAL1 and MTCH2 in the kidney fat and for TFAP2B in the subcutaneous fat. SEC16B was the only gene, which showed the highest expression not in the hypothalamus, but in the subcutaneous fat alone. Concomitant the expression of SEC16B was lowest in the hypothalamus and kidney fat. This fact is in line with recent results from the GIANT consortium, which also found a high expression for a bigger part of the obesity-related genes in the hypothalamus [11] and suggests a critical role of these GWAS obesity associated genes in the central regulation of energy balance.

Despite this fact it is very interesting that there were no significant differences between the target genes in the hypothalamus of ZL and ZDF rats. Differences were observed in the kidney fat, where the expression of LYPLAL1, TFAP2B, NEGR1, SEC16B and FAIM2 was significantly lower in the ZDF rats. Additionally for NEGR1 a significantly lower expression was found in all three adipose tissues of ZDF rats and for MTCH2 a significantly higher expression was found in the subcutaneous and mesenterial fat of ZDF rats. This suggests that putative important mechanisms of the obesity associated genes in the hypothalamus are not responsible for the development of obesity in ZDF rats. Instead changes in the adipose tissue seem to be accountable for the development of the ZDF phenotype.

Taking further into account that ZDF rats exhibit hyperleptinemia (see Table 2) due to a defect in the leptin receptor, we can assume that the actions of those genes without differential expressions between ZL and ZDF rats must be rather independent of leptin levels or its effects.

### Limitations

This study has certain limitations. Our study was planned as a descriptive expression analysis of fourteen novel obesity related genes in the animal model of the ZDF rat. Thereby, the aim was on the one hand to identify tissues, in which those genes are expressed, and on the other hand, to reveal differences in the expression levels between ZDF rats and their littermates the ZL rats at an age of 22 weeks. Therefore, this study does not provide information on alterations in different fasting states of the animals, but this might be of interest for future work considering the fact that a high fat diet is known to induce alterations in metabolism and gene expression of adipose tissues [40]. Furthermore as a sole descriptive study in a certain animal model, our results are not simply assignable to other animal models of obesity. This is particularly because ZDF rats are not only obese, but also develop Typ 2 diabetes mellitus. That is why, we cannot exclude that the observed alterations in the gene expression could at least in part be related to the diabetic state of the animals. Additionally, it has to be mentioned, that ZDF rats are homozygous for a defect in the leptin receptor and it is not clear, whether this alone causes alterations in the expression of the investigated obesity genes. The possible impact of insulin resistance and leptin receptor defect could be further investigated by studying Spontaneously Diabetic Torii (SDT) rats as a model of non-obese type 2 diabetes mellitus and a congenic strain of SDT rat with the same leptin receptor defect as the ZDF rats, which are obese and develop type 2 diabetes mellitus [41].

## Conclusion

Thirteen of our fourteen investigated obesity-related genes showed the highest expression in the hypothalamus suggesting a role of these genes predominantly in the central regulation of energy homeostasis. On the other hand the ZDF phenotype accompanies only with expression changes in the adipose tissues, which implies that peripheral mechanisms are important for the development of obesity in ZDF rats. However, further studies to reveal the exact function of most of these genes are necessary.

## Competing interests

The authors declare that they have no competing interests.

## Authors’ contributions

PS, IH, AL designed research; PS, IH, CB, AS, MR, CB, DE, GR and AL performed research; CB, AS, GR and DE revised the manuscript critically for intellectual content; PS, IH, CB, AS and AL analyzed data; PS and AL wrote the paper. All authors have read and approved submission of the final manuscript.
